# IL-17 and TNF-α Are Key Mediators of *Moraxella catarrhalis* Triggered Exacerbation of Allergic Airway Inflammation

**DOI:** 10.3389/fimmu.2017.01562

**Published:** 2017-11-14

**Authors:** Safa Alnahas, Stefanie Hagner, Hartmann Raifer, Ayse Kilic, Georg Gasteiger, Reinier Mutters, Anne Hellhund, Immo Prinz, Olaf Pinkenburg, Alexander Visekruna, Holger Garn, Ulrich Steinhoff

**Affiliations:** ^1^Institute of Medical Microbiology and Hospital Hygiene, University of Marburg, Marburg, Germany; ^2^Institute of Laboratory Medicine and Pathobiochemistry, Molecular Diagnostics, Member of the German Center for Lung Research, University of Marburg, Marburg, Germany; ^3^Institute of Medical Microbiology and Hygiene, FZI Research Center for Immunotherapy, University of Mainz Medical Center, Mainz, Germany; ^4^Institute of Immunology, Hannover Medical School, Hannover, Germany

**Keywords:** exacerbation of pulmonary inflammation, IL-17, TNF-α, Moraxellaceae infections, infection and allergy, exacerbation of allergic reactions, pulmonary inflammation, microbial exacerbation of pulmonary inflammation

## Abstract

Alterations of the airway microbiome are often associated with pulmonary diseases. For example, detection of the bacterial pathogen *Moraxella catarrhalis* in the upper airways is linked with an increased risk to develop or exacerbate asthma. However, the mechanisms by which *M. catarrhalis* augments allergic airway inflammation (AAI) remain unclear. We here characterized the cellular and soluble mediators of *M. catarrhalis* triggered excacerbation of AAI in wt and IL-17 deficient as well as in animals treated with TNF-α and IL-6 neutralizing antibodies. We compared the type of inflammatory response in *M. catarrhalis* infected, house dust mite (HDM)-allergic and animals infected with *M. catarrhalis* at different time points of HDM sensitization. We found that airway infection of mice with *M. catarrhalis* triggers a strong inflammatory response with massive neutrophilic infiltrates, high amounts of IL-6 and TNF-α and moderate levels of CD4^+^ T-cell-derived IFN-γ and IL-17. If bacterial infection occurred during HDM allergen sensitization, the allergic airway response was exacerbated, particularly by the expansion of Th17 cells and increased TNF-α levels. Neutralization of IL-17 or TNF-α but not IL-6 resulted in accelerated clearance of *M. catarrhalis* and effectively prevented infection-induced exacerbation of AAI. Taken together, our data demonstrate an essential role for TNF-α and IL-17 in infection-triggered exacerbation of AAI.

## Introduction

Constant exposure of the respiratory mucosa to pollutants, allergens, and pathogens requires robust and at the same time strictly regulated immune responses of the airways. A breakdown of controlled airway immune responses to these environmental factors may result in acute or chronic inflammation, including asthma, and chronic obstructive pulmonary disease (COPD). Respiratory tract infections have emerged as the most frequent triggers for pulmonary inflammation in both children and adults (1). Pathogenic bacteria including *Streptococcus pneumoniae, Hemophilus influenzae*, and *Moraxella catarrhalis* are microbial colonizers of the airway mucosa inducing an inflammatory immune response that prepares the ground for increased asthma susceptibility or exacerbation of established disease (2). Infection-triggered airway inflammation is often associated with increased amounts of inflammatory cytokines such as IL-6, IL-17, and TNF-α (3) and infiltrates of neutrophils, eosinophils, and different subtypes of T helper cells (4, 5) which seem to be related to the severity and pathogenesis of pulmonary inflammation (6). Paradoxically, many of these cytokines which induce pulmonary inflammation are also involved in antimicrobial host defense (7, 8), e.g., IL-17 and TNF-α mediate influx of neutrophils mediating first-line defense through uptake and killing of microbes. Although *M. catarrhalis* is a common pathogen known to trigger or exacerbate established pulmonary inflammation, the molecular and cellular mechanism still remains obscure (9).

*M. catarrhalis* is able to efficiently adhere to the epithelium of distinct mucosal tissues such as lung and nasopharynx. Together with *S. pneumoniae* and *H. influenzae, M. catarrhalis* is one of the major pathogens causing otitis media (OM) in children. Furthermore, several studies have reported that *M. catarrhalis* contributes to the exacerbations of COPD in adults (10). During the course of infection, *M. catarrhalis* induces a robust inflammatory response characterized by infiltrations of macrophages, lymphocytes, and neutrophils into infected tissue, which probably causes the pathogenesis of OM and also leads to exacerbations of COPD. The investigation of the molecular interplay between *M. catarrhalis* and host immune system has revealed that the recognition of this pathogen by multiple toll-like receptors (TLRs) such as TLR4 and TLR9 triggers the production of pro-inflammatory cytokines IL-6 and TNF-α (11). In many asthma patients, *M. catarrhalis* is one of the dominant pathogen species found within the airway bacterial community (12). Although a link between infection with *M. catarrhalis* and exacerbation of asthma has been proposed, no functional data exist to our knowledge, indicating that this pathogen is causative for exacerbation of this disease.

Our aim was to investigate whether *M. catarrhalis* infection has the potential to exacerbate allergic airway inflammation (AAI) and if yes, to analyze the underlying pathomechanisms. Therefore, we established for the first time a murine model of *M. catarrhalis* airway infection and rigorously analyzed the mechanisms of pulmonary inflammation triggered exclusively by the pathogen or during developing or established AAI against the house dust mite (HDM) allergen.

We here show that airway infection with *M. catarrhalis* augments the phenotype of AAI mainly *via* TNF-α and T-cell derived IL-17 but not IL-6. Yet, in the absence of IL-17 and/or TNF-α, infection-triggered lung inflammation as well as exacerbation of AAI was very mild and resulted in accelerated clearance of pathogenic airway bacteria, emphasizing the inflammation promoting role of these cytokines.

## Results

### Characteristics of Airway Inflammation Caused by *M. catarrhalis* infection

**Figure 1:**
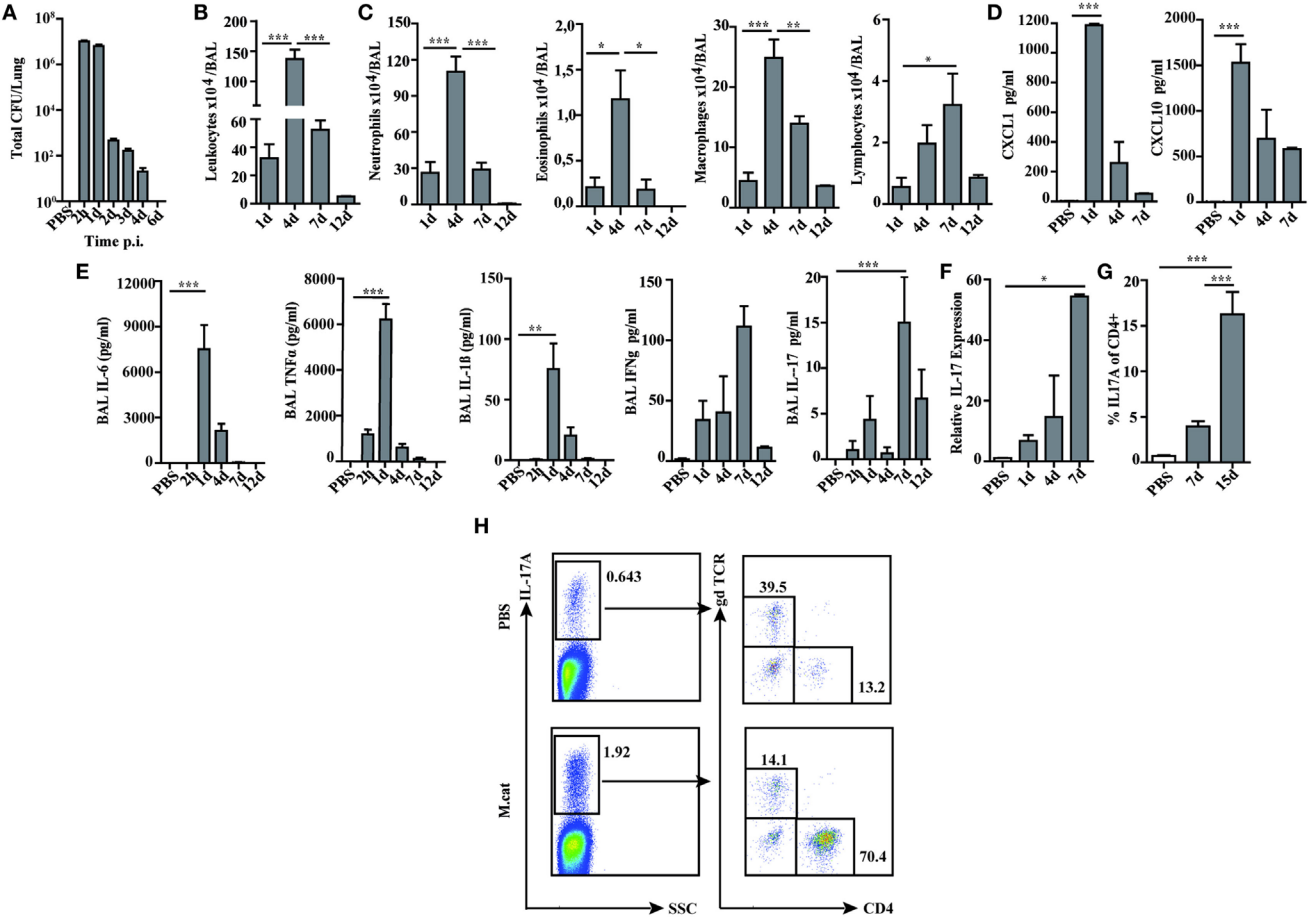
Pulmonary immune response after intranasal *M. catarrhalis* infection. C57Bl/6 animals were infected (i.n.) with 2 × 10^8^ CFU *M. catarrhalis* and BAL and lungs were harvested at indicated time points. **(A)** Bacterial CFU were determined in lung homogenates. **(B)** Total cell counts in BALF. **(C)** Differential cell counts in BALF. **(D)** CXCL1 and CXCL10 chemokines in BALF. **(E)** Cytokines in BALF. **(F)** Quantitative expression of IL-17 mRNA (qPCR) in lungs. **(G)** Frequency of IL-17A^+^CD4^+^ T cells in lungs. **(H)** Analysis of γδ T cells and CD4^+^ T cells secreting IL-17A at day 7 after PBS treatment or infection. *n* = 6 mice per group and two independent experiments were performed. ***P = 0.001, **P = 0.01, and *P = 0.05 (one-way ANOVA). BAL, bronchoaleveolar lavage; BALF, BAL fluid; qPCR, quantitative polymerase chain reaction; PBS, phosphate-buffered saline; ANOVA, analysis of variance.

**Figure 2:**
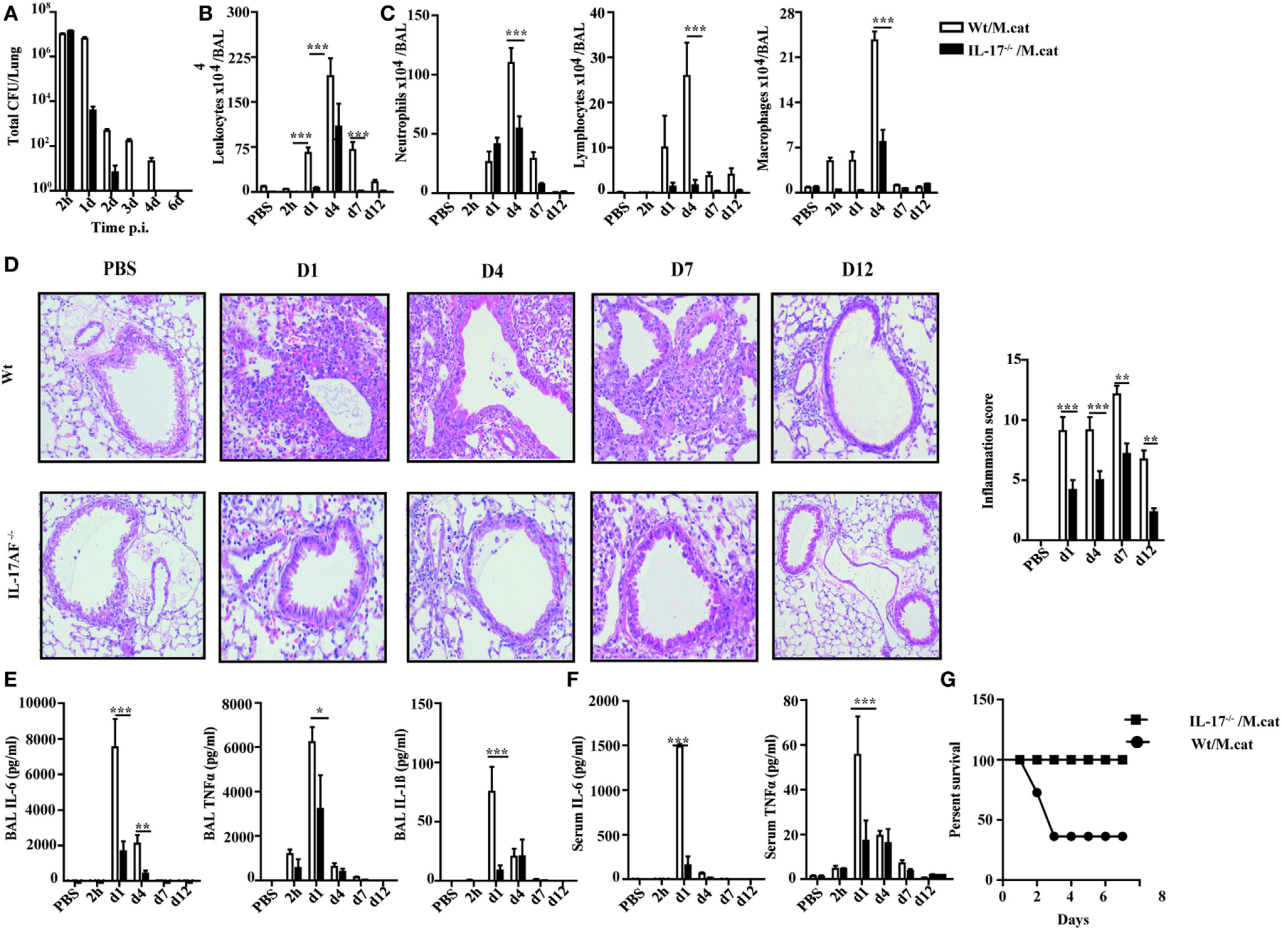
Reduced inflammatory response to *M. catarrhalis* infection in IL-17 KO mice. IL-17 KO (black bars) and wt mice (white bars) were infected (i.n.) with 2 × 10^8^ CFU *M. catarrhalis*. BAL, lungs, and serum were harvested at indicated time points. **(A)** Bacterial CFU in lung homogenates were counted at different time points after infection. **(B)** Airway inflammation represented by BAL total cell counts. **(C)** Differential cell counts in BAL. **(D)** Representative periodic acid Schiff-stained airways of wt and IL-17 KO mice **(E)** Amounts of IL-6, TNF-α, and IL-1β in BALF. **(F)** IL-6 and TNF-α in serum. **(G)** Survival of IL-17 KO (squares) and wt animals (circles) after infection. Data were from two independent experiments (*n* = 10 mice per group). ***P = 0.001, **P = 0.01, and *P = 0.05 (one-way ANOVA). BAL, bronchoaleveolar lavage; BALF, BAL fluid; ANOVA, analysis of variance.

**Figure 3:**
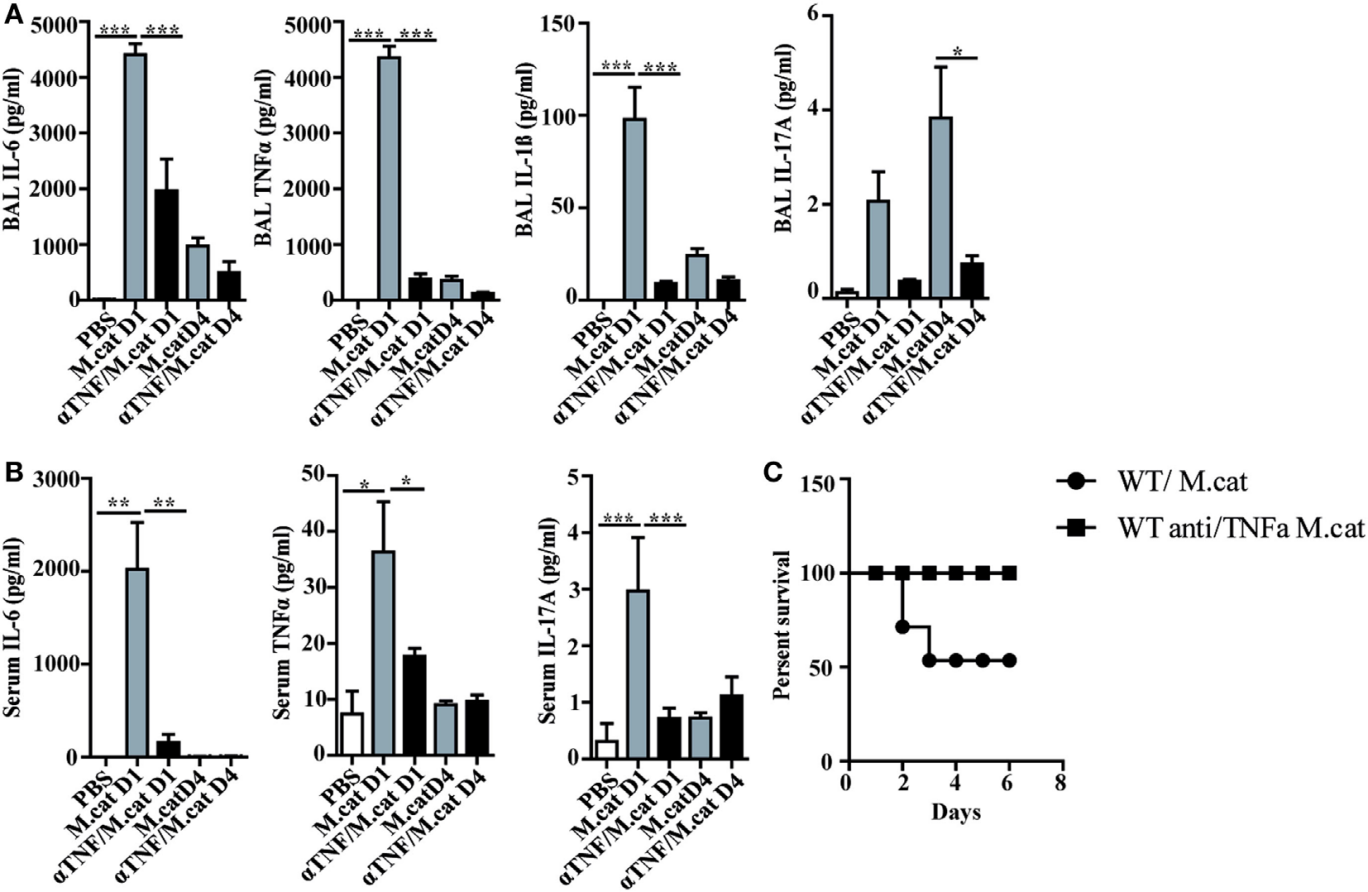
Neutralization of TNF-α protects against respiratory *M. catarrhalis* infection. C57BL/6 animals received a single i.p. injection of 100 µg of anti-TNF-α (black bars) or IgG1 control mAbs (gray bars) and were 4 h later intranasally infected with 2 × 10^8^ CFU *M. catarrhalis*. **(A)** Amounts of IL-6, TNF-α, IL-1β, and IL-17 in BALF of untreated or anti-TNF-α-treated mice at indicated time points. **(B)** As in **(A)** but in serum **(C)** Survival of anti-TNF-α-treated animals (circle) or control IgG-treated (square) mice after infection with *M. catarrhalis*. Data were from two independent experiments, *n* = 6 mice per group. ***P = 0.001, **P = 0.01, and *P = 0.05 (one-way ANOVA). BALF, BAL fluid; ANOVA, analysis of variance.

**Figure 4:**
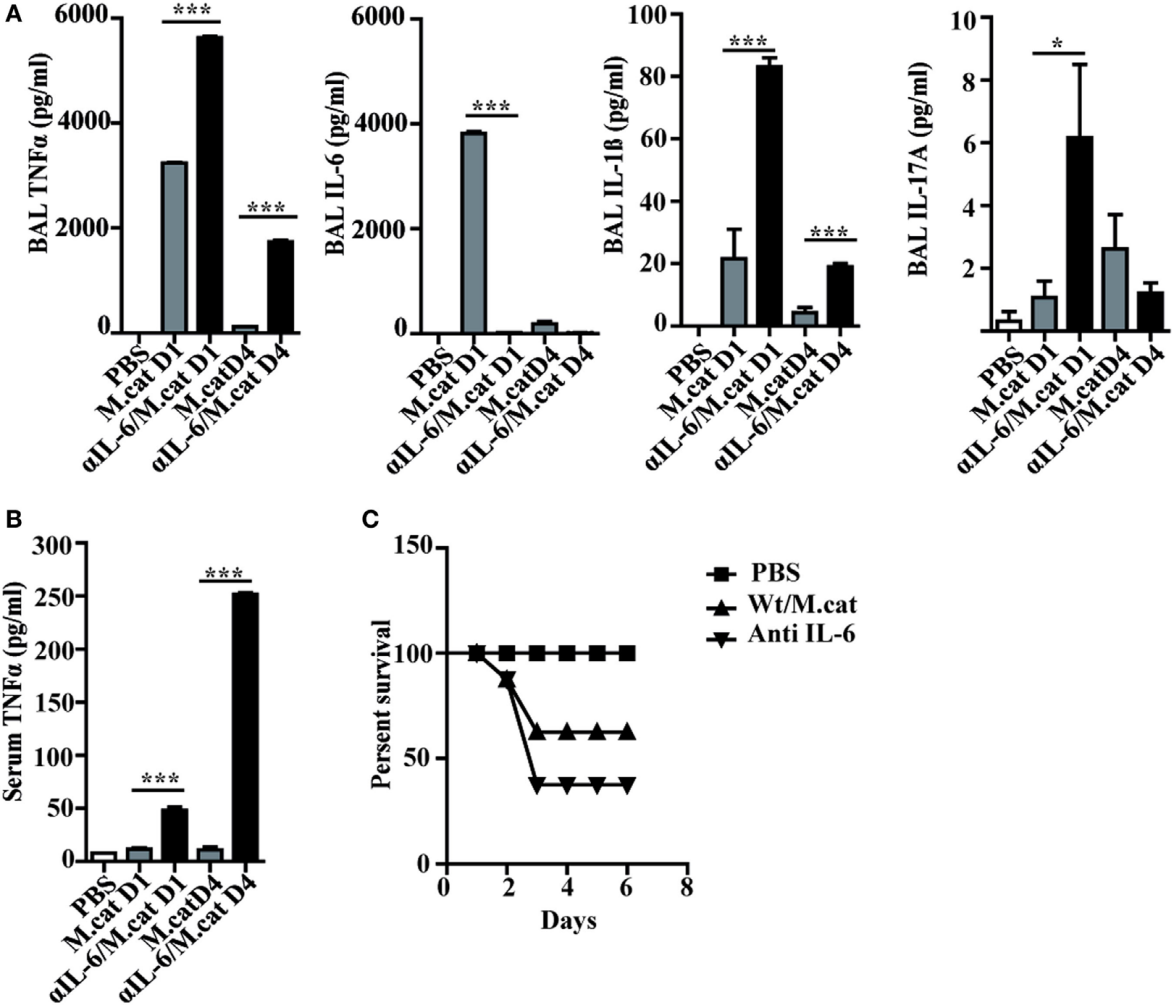
Neutralization of IL-6 enhances *M. catarrhalis* triggered inflammation. C57BL/6 animals received a single i.p. injection of 100 µg of anti-IL-6 or IgG1control mAbs and 4 h later were infected (i.n.) with *M. catarrhalis* (2 × 10^8^ CFU) **(A)** Amounts of TNF-α, IL-6, IL-1β, and IL-17 in BALF. **(B)** Amount of TNF-α in serum. **(C)** Survival of PBS-treated control mice (PBS), control-IgG1-treated animals infected with *M.catarrahlis* (Wt/M.cat) and anti-IL-6-mAb-treated animals infected with *M. catarrhalis* (anti IL-6). Data were from two independent experiments, *n* = 3 mice per group. ***P = 0.001, **P = 0.01, and *P = 0.05 (one-way ANOVA). BALF, BAL fluid; PBS, phosphate-buffered saline; ANOVA, analysis of variance.

Before investigating the consequences of bacterial airway infection on AAI, we established a murine infection model to first study the immune responses triggered by *M. catarrhalis*. Two hours after intranasal inoculation, infection was fully established and bacterial titers of the lung dropped massively at day 2 p.i. and were below detection at day 6, revealing clearance of *M. catarrhalis* in normal animals (Figure 1A). It has to be noted that approx. 50% of wt animals intranasally infected with *M. catarrhalis* showed moribund symptoms at day 3 p.i. (Figure 2G). Total cell numbers in bronchoaleveolar lavage (BAL) increased 1 day after intranasal inoculation, peaked at day 4, and then decreased. *M. catarrhalis* infection caused airway inflammation primarily through massive influx of neutrophils and lower numbers of eosinophils and macrophages reaching their maximum at day 4, followed by an increase in lymphocytes which peaked at day 7 p.i. Twelve days after infection, all BAL cells numbers returned to baseline level (Figures 1B,C). Accordingly, early and strong expression of CXCL1 and CXCL10 in the BAL was observed, both known to attract neutrophils and other inflammatory cells (Figure 1D). As *M. catarrhalis* infection causes airway inflammation (9, 13), we performed a kinetic study of inflammatory cytokines in the BAL. While IL-6, TNF-α and IL-1β peaked at day 1 p.i. and rapidly decreased to low levels by day 4, the amounts of IFN-γ and IL-17 were highest at day 7 p.i., coinciding with the peak of BAL lymphocytes (Figure 1E). Due to the late occurrence of IL-17 in the BAL, we continued to analyze the mRNA expression of this cytokine in lung homogenates. In accordance with BAL data, strong IL-17 mRNA expression was found at day 7, correlating with the rise of IL-17^+^CD4^+^ T cells at this time point (Figures 1F,G). To identify the type of T cells producing pulmonary IL-17 after *M. catarrhalis* infection, lung lymphocytes were analyzed by flow cytometry. The majority of IL-17 secreting cells in the lung were conventional CD4^+^ T cells (70%) which increased between days 7 and 15 p.i. and only a minor fraction of TCR γ^+^δ^+^ cells (14%) was positive for IL-17 (Figure 1H). We next investigated inflammatory responses in IL-17 deficient mice. Interestingly, despite similar initial bacterial colonization of the lungs, IL-17 KO mice showed increased bacterial clearance as compared to wt animals (Figure 2A). Furthermore, these mice revealed significantly reduced total and differential BAL cell counts (Figures 2B,C) and lung tissues showed a massive reduction of cellular infiltrates as compared to wt animals (Figure 2D). The amounts of IL-6 and TNF-α in BAL and serum of infected IL-17 KO were diminished and mice did not succumb to *M. catarrhalis* infection wt animals (Figures 2E–G). Interestingly, despite the lack of IL-17, the number of neutrophils in the lung at day 1 p.i. was comparable to wt animals. Similar amounts of the CXCL1, 5, and 10 chemokines in IL-17 KO mice might compensate for early neutrophil attraction, independently in IL-17 (Figure S1 in Supplementary Material). Likewise, neutralization of TNF-α, which is also produced by epithelial cells upon *M. catarrhalis* infection (Figure S2 in Supplementary Material), resulted in reduction of local and systemic IL-1β, IL-6, and IL-17 and subsequent protection against *M. catarrhalis* infection (Figures 3A–C). Interestingly, in contrast to IL-17 and TNF-α, we found that neutralization of IL-6 increased local and systemic TNF-α as well as early IL-1β and IL-17 levels in the BAL. Anti-IL-6 treatment resulted in slightly enhanced numbers of animals with moribund symptoms, suggesting that IL-6 exerts an anti-inflammatory function during pulmonary *M. catarrhalis* infection (Figures 4A–C).

### Pulmonary Infection with *Moraxella catarrhalis* Exacerbates HDM-Induced AAI

**Figure 5:**
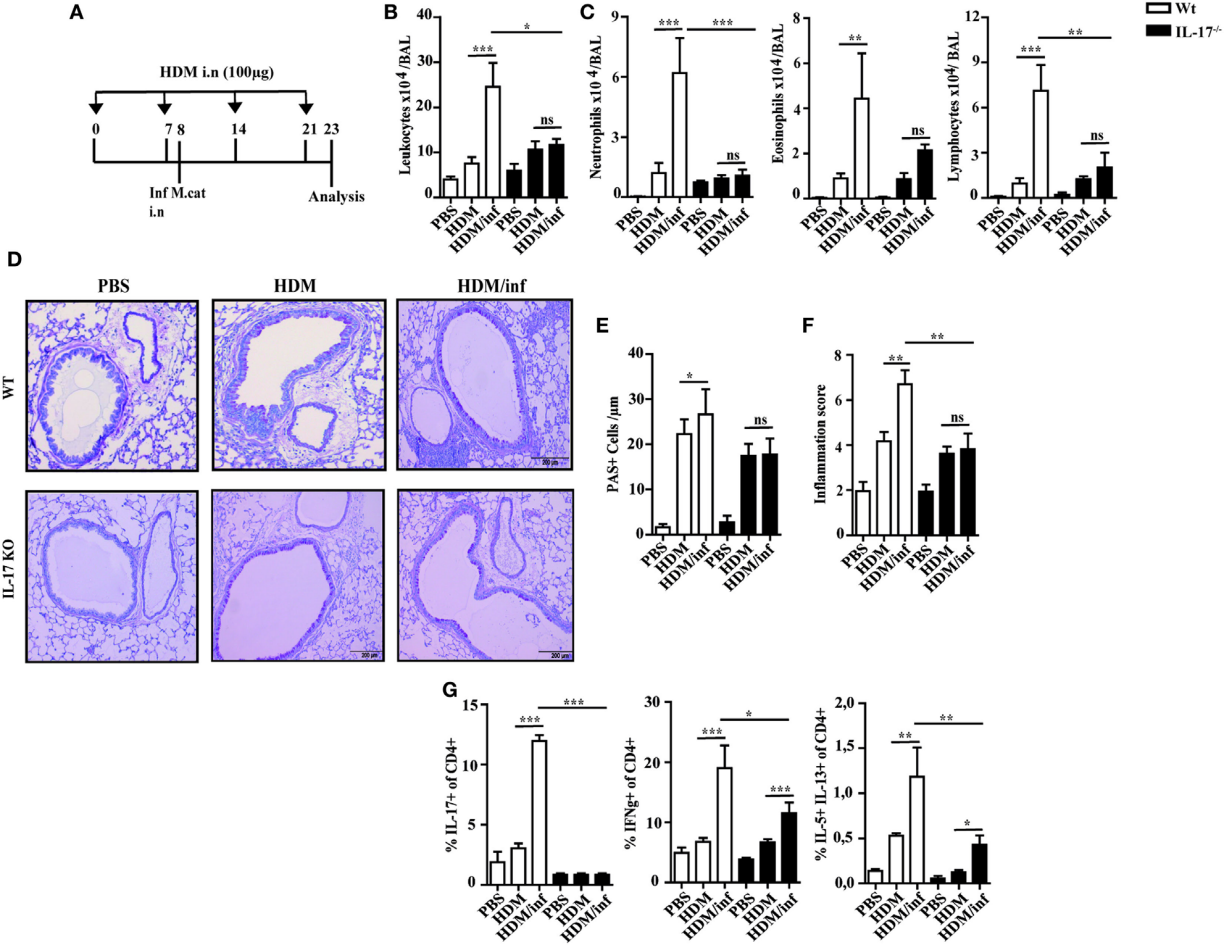
IL-17 is a key cytokine of *M. catarrhalis*-induced exacerbation of AAI. C57BL/6 (white bars) and IL-17 KO animals (black bars) were infected (i.n.) with 2 × 10^8^ CFU *M. catarrhalis* after the second HDM exposure. BAL and lungs were analyzed at day 23. **(A)** Protocol. **(B)** Total cell counts in BAL. **(C)** Differential cell counts. **(D)** Representative periodic acid Schiff-stained airways. **(E)** Goblet cell counts in lung tissues. **(F)** Inflammation score. **(G)** Intracellular staining of CD4^+^ lung T cells for IL-17A, IFN-γ, IL-5, and IL-13. Data were from two independent experiments, *n* = 8 mice per group. ***P = 0.001, **P = 0.01, and *P = 0.05 (one-way ANOVA). AAI, allergic airway inflammation; HDM, house dust mite; BAL, bronchoaleveolar lavage; ANOVA, analysis of variance.

To study the mechanisms of *M. catarrhalis* caused exacerbation of AAI, wt C57Bl/6 mice (white bars) were intranasally infected with *M. catarrhalis* after the second exposure with HDM and analyzed on day 23 as shown in Figure 5A. While HDM exposure induced airway inflammation, additional infection of HDM sensitized wt mice with *M. catarrhalis* led to hugely increased numbers of leukocytes in the BALF mainly through infiltrating neutrophils, eosinophils, and lymphocytes (Figures 5B,C). This was also evident in lung histology, showing increased airway inflammation, goblet cell hyperplasia, and mucus production in *M. catarrhalis* infected, HDM-allergic mice (Figures 5D–F). To further study the cytokine pattern of pulmonary CD4^+^ T-cell populations, we compared HDM allergic mice with those that additionally contracted an infection. The latter group revealed significantly increased frequencies of IFN-γ, IL-17A, and IL-5^+^/IL-13^+^ double positive CD4^+^ T cells, although the absolute frequencies of IL-5^+^/IL-13^+^ Th2 cells were low as compared to those of IFN-γ^+^ and IL-17^+^CD4^+^ T cells (Figure 5G; Figure S5A in Supplementary Material). Accordingly, increased amounts of IL-17 and IFN-γ were found in the lungs of HDM allergic wt mice (white bars) concomitantly infected with *M. catarrhalis* (Figure S5B in Supplementary Material). Of note, cellular infiltrates and cytokine responses measured at day 23 were significantly lower than during acute infection with *M. catarrhalis*.

We then wondered whether the time of *M. catarrhalis* infection (prior to, during or after complete HDM sensitization) has an influence on exacerbation of AAI. Similar to infection during HDM sensitization, bacterial infection after established allergic reactions increased total BAL leukocyte counts mainly by neutrophils and lymphocytes. Together with increased lymphocytic infiltration, airway infection enhanced the frequencies of lung IL-17, IFN-γ, and IL-5 secreting CD4^+^ T cells as compared to with the HDM-only group (Figure S3 in Supplementary Material). Furthermore, we wondered whether a previously resolved infection with *M. catarrhalis* is still able to exacerbate HDM allergic reactions. Similar to infection during or after HDM sensitization, infection prior to allergen exposure triggered airway infiltrations of CD4^+^ T cells secreting increased amounts of IL-17, IFN-γ, and IL-5 (Figure S4 in Supplementary Material).

### IL-17 Is a Central Mediator of Infection-Triggered Exacerbation of AAI

Since IL-17 has been reported to be associated with mucoepithelial infections and allergic responses (14), we next tested IL-17 deficient mice for the development of HDM-allergic airway responses. Interestingly, wt animals (white bars) and IL-17 KO mice (black bars) revealed similar numbers of neutrophils, eosinophils, and lymphocytes in the BALF (Figures 5B,C). Lung histology revealed slightly reduced pulmonary infiltrates in IL-17 KO mice as compared to with wt mice after HDM exposure (Figure 5D). Accordingly, airway inflammation, goblet cell hyperplasia, and mucus production were marginally diminished in IL-17 KO mice as compared to with controls (Figures 5E,F).

As early antibacterial immunity is mediated by IL-17-controlled influx of neutrophils into infected tissues (15), we next investigated the role of this cytokine to trigger infection-induced exacerbation of AAI. IL-17 KO and wt mice were infected during the second HDM allergen exposure and analyzed at day 23, as shown in Figure 5A. The data demonstrate that in contrast to wt animals, the number of neutrophils, eosinophils, and lymphocytes in the BALF of infected IL-17 KO mice was comparable to the non-infected, HDM group. Furthermore, comparable numbers of mucus secreting cells and similar inflammation scores suggest IL-17 as key cytokine of *M. catarrhalis*-induced exacerbation of AAI (Figures 5D–F). Yet, *M. catarrhalis* infection of HDM allergic IL-17 KO mice still enhanced the frequency of pulmonary IFN-γ and IL-5/IL-13 secreting CD4^+^ T cells (Figure 5G; Figures S5A,B in Supplementary Material).

Taken together, these findings show that IL-17 essentially contributes to *M. catarrhalis* triggered exacerbation of AAI.

### Anti TNF-α Treatment Attenuating IL-17-Dependent Exacerbation of Infection-Induced AAI

**Figure 6:**
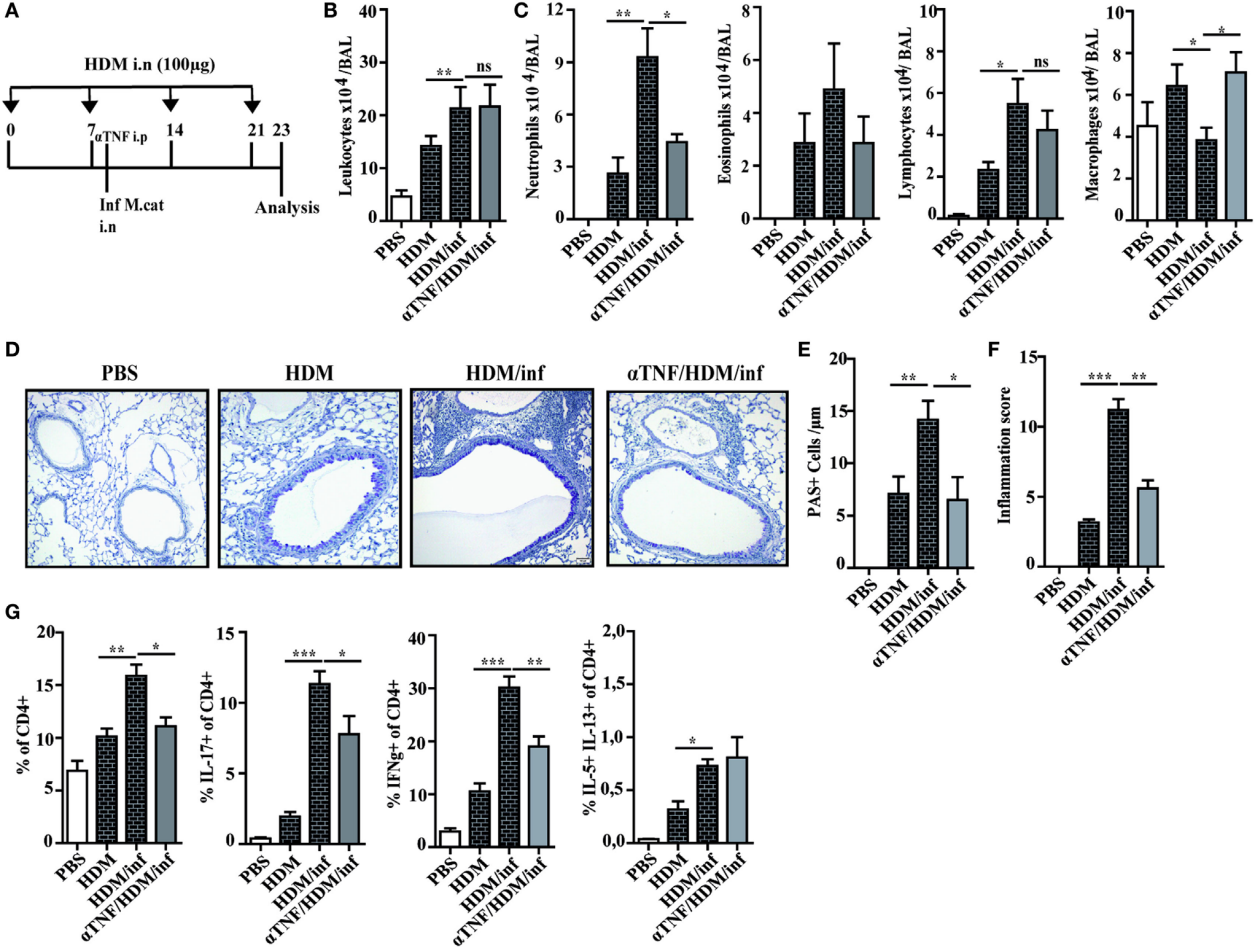
Anti TNF-α treatment alleviates *M. catarrhalis*-induced exacerbation of HDM AAI. C57BL/animals were infected (i.n.) with 2 × 10^8^ CFU *M. catarrhalis* after the second HDM exposure. Mice were administered a single dose of 100 µg anti TNF-α or IgG1 control mAb 4 h prior to *M. catarrhalis* infection. BAL and lungs were analyzed at day 23. **(A)** Protocol **(B)** Total cell counts in BAL. **(C)** Differential cell counts. **(D)** Representative periodic acid Schiff-stained airways. **(E)** Goblet cell counts in lung tissues. **(F)** Inflammation score. **(G)** Intracellular staining of lung CD4^+^ T cells for IL-17A, IFN-γ, and IL-5/IL-13 positive cells. Data were from two independent experiments, *n* = 8 mice per group. ***P = 0.001, **P = 0.01, and *P = 0.05 (one-way ANOVA). HDM, house dust mite; AAI, allergic airway inflammation; BAL, bronchoaleveolar lavage; ANOVA, analysis of variance.

It has been shown that neutrophil recruitment during inflammation is mediated by synergistic effects of IL-17 and TNF-α in endothelial cells by enhancing the expression of P- and E-selectin (16). We thus wondered whether neutralization of TNF-α also attenuates infection-induced exacerbation of AAI. Briefly, HDM sensitized mice were treated only once with anti-TNF-α mAb 4 h before intranasal infection with *M. catarrhalis* and analyzed at day 23, as depicted in Figure 6A. Control groups included mice that received phosphate-buffered saline (PBS) or HDM alone as well as infected, HDM sensitized animals.

Differential cell counts of BAL cells revealed that predominantly neutrophils were reduced by anti-TNF-α treatment, while the numbers of macrophages were increased when compared with HDM/infected controls. The increase of BAL macrophages after TNF-α neutralization seems to compensate for the low number of neutrophils in order to eliminate *M. catarrhalis*. Interestingly, the number of eosinophils or lymphocytes were not significantly reduced after TNF-α treatment as shown in Figures 6B,C. Lung histology confirmed that anti-TNF-α treatment vastly reduced infection-triggered inflammatory infiltrates in HDM allergic animals which was also reflected by the decreased number of PAS^+^ cells and the low-inflammation score (Figures 6D–F). We next wondered whether TNF-α neutralization also affects pulmonary T-cell activation. Anti-TNF-α treatment attenuated the activation of IFN-γ^+^ and IL-17^+^ lymphocytes but had no influence on the low frequency of IL-5/IL-13 secreting, pulmonary CD4^+^ T cells (Figure 6G; Figure S6 in Supplementary Material).

In summary, the experiments revealed that a single application of anti-TNF-α mAb is sufficient to markedly attenuate *M. catarrhalis*-induced exacerbation of AAI.

## Discussion

We here show that infection with *M. catarrhalis* exacerbates HDM-triggered AAI mainly by an IL-17- and TNF-α-dependent inflammatory response. Lack of IL-17 or neutralization of TNF-α prevented infection-triggered exacerbation of allergic pulmonary disease.

Infection with *M. catarrhalis* has been shown to be an important risk factor for newborns or patients with chronic pulmonary diseases to either develop asthma or to exacerbate disease symptoms (17). While evidence suggests that the frequency of respiratory infections rather than the type of pathogen influences asthma development (15), the immunological mechanisms of infection-triggered exacerbation of AAI are not entirely understood, which is reflected by the limited therapeutic options.

Infection with *M. catarrhalis* during HDM allergen exposure strongly increased the influx of neutrophils, eosinophils, and T cells, while infection after established allergic inflammation caused exacerbation primarily by neutrophils. In contrast, infection prior to HDM exposure predominantly triggered the expansion of pulmonary T-lymphocyte populations. However, common to all three settings of infection is the induction of a mixed pulmonary Th1, Th2, and Th17 immune response. Thus, our data suggest that bacterial infection influences the development and outcome of AAI irrespective of the time-point of allergen exposure. This reflects the situation in patients where viral and/or bacterial infections in early life or during asthma have been associated with the development or exacerbation of the disease, respectively (18).

The importance of IL-17 in protection against pulmonary bacterial infection was shown for several pathogens, including *Klebsiella pneumoniae, Mycoplasma pneumoniae, Bordatella pertussis*, and *Mycobacterium tuberculosis* (19). Accordingly, IL-17 receptor KO mice revealed increased susceptibility to bacterial infections due to impaired neutrophil and macrophage recruitment to the site of bacterial entry (20). On the other hand, the expression of IL-17 in the airways of asthma patients was shown to correlate with neutrophilic lung inflammation (21–23). Despite this ambiguous role, our data revealed that IL-17 deficient animals cleared the pathogen even faster than control littermates. Unimpaired expression of CXCL1, 5, and 10 chemokines in IL-17 KO mice seems to compensate for early neutrophil influx but not for later stages of infection where the pulmonary neutrophil response was decreased in KO animals.

Infection of human respiratory tract epithelial cells with *M. catarrhalis* has been shown to increase various pro-inflammatory genes, e.g., TNF-α, IL-1β, and IL-17 (10). We observed a similar cytokine response in *M. catarrhalis* infected wt but not IL-17 KO mice which showed even increased resistance against infection. Furthermore, in the absence of IL-17, the mixed neutrophilic/eosinophilic airway inflammation of HDM challenged wt mice switched to an attenuated, almost exclusive eosinophilic response. Likewise, *M. catarrhalis* infection of HDM allergic mice did not lead to exacerbation of pulmonary inflammation in IL-17 deficient mice. Taken together, these findings indicate that pulmonary infection is a crucial trigger for IL-17-induced, exacerbation of neutrophilic inflammation.

Besides IL-17, IL-6 has been considered as promising target to reduce pathogenesis of asthma and COPD, particularly those of eosinophilic/neutrophilic phenotype (24, 25). IL-6 is known to mediate pro-inflammatory and anti-inflammatory immune functions *via* trans- and classical signaling, respectively (26). As anti-IL-6 treatment led to enhanced secretion of local and systemic TNF-α with high susceptibility to *M. catarrhalis* infection, we did not consider IL-6 as appropriate target for treatment.

The contradicting reports about efficiency and safety of TNF-α blockers in animal models and patients with severe asthma (27, 28) prompted us to study the effect of TNF-α neutralization during pulmonary infection and exacerbation of AAI.

We found that neutralization of TNF-α prior to pulmonary *M. catarrhalis* infection resulted in a substantial decline of IL-6, Il-1β, and IL-17, together with increased survival of infected animals. Furthermore, TNF-α neutralization was able to effectively reduce infection-induced exacerbation of HDM-allergic reactions despite minor or no reduction of pulmonary Th1, Th17, and Th2 cells, respectively. Even though we found a strong decrease of neutrophils in the BAL, the mechanisms by which TNF-α acts in asthmatic airways might be manifold: Besides its effect to recruit neutrophils and eosinophils (29), it has been shown to enhance T-cell responses (30), to promote glucocorticoid resistance and airway remodeling (31).

While it has been shown that TNF-α or IL-17 alone are able to induce inflammatory responses, inflammation was enhanced in the presence of both cytokines (32, 33).

In line with these observations, our data demonstrate that neutralization of IL-17 or TNF-α is similar effective in attenuating exacerbation of AAI. Whether neutralization of these cytokines is also suitable for therapy still remains to be determined. Since prolonged anti TNF-α or IL-17 therapies bear the risk of severe infections or development of metaplastic malignancies (28, 34, 35), only short-term treatment with combined anti-IL-17/anti-TNF-α antibodies might be qualified for the treatment of infection-triggered exacerbation of AAI.

## Materials and Methods

### Animals

Female 8–10-weeks-old C57BL/6J mice were purchased from Charles River Laboratories, Germany. Female IL-17AF^−/−^ mice on C57BL/6J background were kindly provided by Prof. Dr. Immo Prinz (Hannover, Germany) and housed in a pathogen-free facility with individually ventilated cages. All experiments were approved by the Regierunspräsidium Gießen (Protocol no. MR 20/6 Nr. 123/2012).

### Bacteria and Infection

A patient isolate of *M. catarrhalis* was stored at −80°C in glycerol. Bacteria from frozen stocks were grown aerobically in Columbia blood agar plates (BBL; Becton Dickinson, MD, USA) with 5% sheep blood at 37°C overnight, washed off the plate and resuspended in sterile PBS for infection. Anesthetized mice were inoculated intranasally (i.n.) with 2 × 10^8^ CFU *M. catarrhalis* resuspended in 50-µL PBS, before during or after HDM challenge.

### Determination of CFU in Organs

Lungs were aseptically removed and homogenized in 1 mL of sterile PBS.

Serial dilutions of BAL fluid and lung homogenates were prepared in sterile PBS, plated on Columbia blood agar plates (BBL; Becton Dickinson, MD USA) with 5% sheep blood and incubated overnight at 37°C. Colonies were enumerated and bacterial numbers per lung calculated.

### Experimental Model of Acute HDM AAI

8–10-week-old C57Bl/6 mice were slightly anesthetized (Ketamin/2%Rompun, i.p.) and weekly challenged by intranasal (i.n.) administration of 100-µg HDM extract (GREER, Lenoir, USA) in 50-µL PBS on days 0, 7, 14, and 21. Two days after last HDM challenges mice were sacrificed and blood, bronchoalveolar lavage (BAL) fluids, lungs were collected for further analysis.

### Administration of Neutralizing Antibodies

To assess the role of IL-6 and TNF-α during the infection with *M. catarrhalis*, neutralizing anti-IL-6 (clone MP5-20F3) or anti-TNF-α (clone MP6-XT22) antibodies were used. Rat IgG1 isotype control was applied for treatment of control mice. Four hours before *M. catarrhalis* infection, mice were *treated intraperitoneally* once with 100 µg of neutralizing antibodies, respectively. All used antibodies were generated and purified by Manuela Staeber (Max Planck Institute for Infection Biology, Berlin, Germany).

### Bronchoaleveolar Lavage

Bronchoaleveolar lavage was performed 48 h after the last challenge using 1-mL cold PBS supplemented with a protease inhibitor cocktail (Roche, Mannheim, Germany). An automated Casy TT cell counter (Schaerfe System, Reutlingen, Germany) was used to determine the total leukocyte cell counts. Cells were centrifuged and the cell-free supernatant was stored at −20°C until cytokines were measured by Elisa. For differential cell counts, Cytospin preparations were fixed and then stained with Diff-Quick (Merz &Dade AG, Dudingen, Switzerland). Macrophages, lymphocytes, eosinophils, and neutrophils were identified by standard morphologic criteria and 300 cells were counted per cytospin. The measurement of cytokines IFN-γ, IL-17, and chemokines (CXCL1, CXCL5, and CXCL10) was performed by Cytometric Bead Array (BD, Heidelberg, Germany) according to manufacturer’s protocol.

### Histology of the Airways

Directly after BAL, lungs were fixed with 10% formalin *via* the trachea, removed, and stored in 10% formalin. Lung tissues were embedded into paraffin. Tissues were cut in 3-µm sections and stained using hematoxylin and eosin (HE) as well as periodic acid Schiff (PAS). Goblet hyperplasia was assessed by cell counting and expressed as the number of goblet cells per 100-µm basement membrane, according to Foster et al. (2000).

### Measurement of HDM-Specific Antibodies in Serum Samples

The amount of HDM-specific IgG1 and IgG2c antibodies was measured by the ELISA. Maxi-Sorb plates were coated with 50-µg/mL HDM extract in bicarbonate buffer (pH 9.6) overnight at 4°C. Subsequently, coated wells were blocked with 1% w/V BSA in PBS for 2 h at RT. After washing, serum samples (1:100) were incubated overnight at 4°C, washed, and incubated with the corresponding biotin-labeled anti-immunoglobulin antibody overnight at 4°C. Plates were washed and incubated with streptavidin-peroxidase for 30 min at RT. After development with substrate, the reaction was stopped using H2SO4 and ODs were read at 450 nm.

### Measurement of Cytokines in BAL Fluid (BALF)

TNF-α, IL-6, IL-1β, IL-5, IL-13, IFN-γ, and IL-17 were measured in cell-free lavage fluid by Cytometric Bead array (CBA; BD BIO-sciences; San Diego, CA, USA) as described by the manufacturer.

### Quantitative Analysis of Cytokine Expression

Expression of IFN-γ and IL-17 mRNA was determined by quantitative real-time polymerase chain reaction (RT-PCR) analysis using LightCycler technology (Roche). RNA was extracted from lung tissues according to manufacturer’s instructions. cDNA synthesis was performed after removal of contaminating genomic DNA by DNase treatment using Superscript reverse transcriptase (Invitrogen, Karlsruhe, Germany) as described by manufacturer. Quantitative PCR (QPCR) was performed by using the QuantiTect SYBR Green PCR kit (QIAgen) and primers specific for IFN-γ (forward: 5′ GCT TTG CAG CTC TTC CTC AT 3′ and reverse 5′ GCA GGA TTT TCA TGT CAC CA 3′), IL-17 (forward: 5′ AAG GCA GCA GCA ATC ATC CC 3′ and reverse 5′ GGG TCT TCA TTG CGG TGG AG 3′) Results were expressed as *x*-fold expression in relation to the house keeping gene L-32.

### Flow Cytometry

Lungs were cut into 2–5 mm pieces and incubated for 30 min at 37°C in the incubation medium (IM) (RPMI-1640 with L Glutamine and NaHCO3, 10% FCS, 16 non-essential amino acids (PAA, Pasching, Austria), 100 mg/mL streptomycin, 120 mg/mL penicillin) supplemented with 1-mg/mL Collagenase D (Roche, Basel, Switzerland) and 20 µg/mL DNase I (Roche, Basel, Switzerland). The pre-digested lungs were minced through 100-mm nylon cell strainer (BD Falcon, NJ, USA) diluted with IM and centrifuged at 1,700 rpm for 5 min. Cell pellets were resuspended in erythrocytes lysis buffer (8 g/L NH_4_Cl; 1 g/L KHCO_3_; 37.2 mg/L EDTA) and incubated at room temperature for 3–5 min. Cells were washed, centrifuged, and resuspended in IM supplemented with 50 ng/mL PMA, 750 ng/mL ionomycin, and 10 mg/mL brefeldin A for 4 h. Extracellular staining was performed in PBS with 1% FCS and presence Fc blocking antibody (clone 2.4G2, BD, Heidelberg, Germany) and fluorophore-labeled antibodies (eBioscience, San Diego, CA, USA, if not specified otherwise). The following antibodies for extracellular T-cell staining was performed: anti-CD4-V450 (RM4-5), anti-CD8α-V500 (53-6.7), and anti-CD25-PE-Cy7 (PC61.5) (BD, Heidelberg, Germany). After extracellular staining, T cells were fixed with FoxP3-Fixation Kit (eBioscience, San Diego, CA, USA) and permeabilized with 0.3% Saponin, 1% FCS in PBS followed by an intracellular staining with the following fluorophore-labeled antibodies: IFN-γ-PerCP-Cy5.5 (XMG1.2, Biolegend, San Diego, CA, USA); IL-17-AlexaFluor 700 and FoxP3-AlexaFluor 700 (FJK-16 s); IL-5-PE (TRFK5); IL-13-AlexaFluor 647 [(eBio13A) (eBioscience, San Diego, CA, USA)]. Every staining included negative and isotype controls. Fluorescence signals were acquired by flow cytometry (FACSAria III; BD, Heidelberg, Germany) and analyzed using FACSDivaTM software.

### Statistical Analysis

Data are expressed as ± SEM. We performed one-way analysis of variance (ANOVA) statistical analysis followed by Bonferroni HSD test using GraphPad Prism 5 software. The values of *P* < 0.05 were considered to be significant.

## Ethics Statement

This manuscript does not include human studies.

## Author Contributions

US designed the study, evaluated the data, and wrote the manuscript. SA performed the experiments and evaluated data. SH contributed to the design of experiments. HR contributed to the design of experiments and evaluated Facs data. AK critically read the manuscript and evaluated data. RM organized and identified microbial material and discussed findings. IP contributed animals, evaluated data, and critically read the manuscript. OP performed experiments. AV evaluated data and critically read the manuscript. HG advised experiments, read the manuscript, and evaluated the data. AH contributed with experiments and GG advised experiments and evaluated Facs data.

## Conflict of Interest Statement

The authors declare that the research was conducted in the absence of any commercial or financial relationships that could be construed as a potential conflict of interest.

## Supplementary Material

Figure S1 Early neutrophil influx induced by *Moraxella catarrhalis* is independent of IL-17. WT and IL-17AF^−/−^ mice were infected (i.n.) with 2 × 10^8^ CFU *M. catarrhalis*. BALF and lungs were harvested at indicated time points. **(A)** Identification of BALF neutrophils by flow cytometry. Neutrophils were stained with Ly6C^+^Iy6G^+^ cells on day one after infection. **(B)** Amounts of CXCL1, CXCL5, and CXCL10 chemokines in BALF, *n* = 5 mice per group. Two independent experiments were performed ****P* = 0.001, ***P* = 0.01, and **P* = 0.05 (one-way ANOVA). BALF, BAL fluid; ANOVA, analysis of variance.

Figure S2 Tracheal epithelial cells produce TNF-*a* upon *Moraxella catarrhalis* infection. Primary tracheal cells from C57BL/6 mice were cultured and left untreated or infected with indicated MOI of *M. catarrhalis*. Culture supernatants were taken at indicated time-points and MOI and analyzed for secretion of TNF-α. Data represent two independent experiments with triplicates for each treatment condition. ****P* = 0.001, ***P* = 0.01, and **P* = 0.05 (one-way ANOVA). ANOVA, analysis of variance.

Figure S3 *Moraxella catarrhalis* exacerbates established HDM-allergic inflammation. C57BL/6 animals were infected (i.n.) with 2 × 10^8^ CFU *M. catarrhalis* after the last HDM exposure at day 22. BAL and lungs were analyzed at day 29. **(A)** Protocol. **(B)** Total cell counts in BALF. **(C)** Differential cell counts. **(D)** Representative periodic acid Schiff-stained airways. **(E)** Goblet cell counts in lung tissues. **(F)** Inflammation score. **(G)** Intracellular staining of lung CD4^+^ T cells for IL-17A, IFN-γ, and IL-5. Data were from two independent experiments (*n* = 8 mice per group). ****P* = 0.001, ***P* = 0.01, and **P* = 0.05 (one-one-way ANOVA). HDM, house dust mite; BAL, bronchoaleveolar lavage; BALF, BAL fluid; ANOVA, analysis of variance.

Figure S4 *Moraxella catarrhalis* infection prior to HDM exposure enhances pulmonary T-cell responses. C57BL/6 animals were infected (i.n.) with 2 × 10^8^ CFU *M. catarrhalis*. Seven days prior to HDM exposure. BAL and lungs were analyzed at day 23. **(A)** Protocol. **(B)** Total cell counts in BALF. **(C)** Differential cell counts. **(D)** Representative FACs analysis of pulmonary CD4^+^ T cells stained for IL-17A, IFN-γ and IL-5/IL-13. **(E)** Statistical distribution of T-cell populations (eight animals) corresponding to the analysis in D. Data were from two independent experiments, *n* = 4 mice per group. ****P* = 0.001, ***P* = 0.01, and **P* = 0.05 (one-way ANOVA). HDM, house dust mite; BAL, bronchoaleveolar lavage; BALF, BAL fluid; ANOVA, analysis of variance.

Figure S5 Analysis of cytokines in the lung of mice infected with *Moraxella catarrhalis* during HDM exposure. C57BL/6 and IL-17 KO animals were infected (i.n.) with 2 × 10^8^ CFU *M. catarrhalis* after the second HDM exposure. Lungs were analyzed at day 23. **(A)** Intracellular staining of lung CD4^+^ T cells was performed for IL-17A, IFN-γ, IL-5, and IL-13. Dot plots are representative of three similar experiments. **(B)** Amounts of IFN-γ, IL-17, IL-5, and IL-13 were measured by ELISA in lung homogenates. Data were from three independent experiments (*n* = 8 mice per group). ***P* = 0.01 and **P* = 0.05 (one-way ANOVA). HDM, house dust mite; ANOVA, analysis of variance.

Figure S6 Effects of anti-TNF-α on inflammatory cytokines production in *M. catarrhalis* infected, HDM allergic mice. C57BL/6 animals were infected (i.n.) with *M. catarrhalis* (2 × 10^8^ CFU) after the second HDM exposure. Mice were treated i.p. with 100-µg anti-TNF-α or IgG1 control mAb, 4 h before infection with *M. catarrhalis*. Lung lymphocytes were analyzed at day 23. **(A)** Intracellular staining of lung CD4^+^ T cells for IL-17A, IFN-γ, IL-5, and IL-13. Data were from two independent experiments (*n* = 16 mice per group). ****P* = 0.001, ***P* = 0.01, and **P* = 0.05 (one-way ANOVA). HDM, house dust mite; ANOVA, analysis of variance.
