# Supplementary figures and images for: IL-17 and TNF-α Are Key Mediators of Moraxella catarrhalis Triggered Exacerbation of Allergic Airway Inflammation
Source: Front Immunol. 2017 Nov 14;8:1562. doi: 10.3389/fimmu.2017.01562 (PMC5694487; doi:10.3389/fimmu.2017.01562)

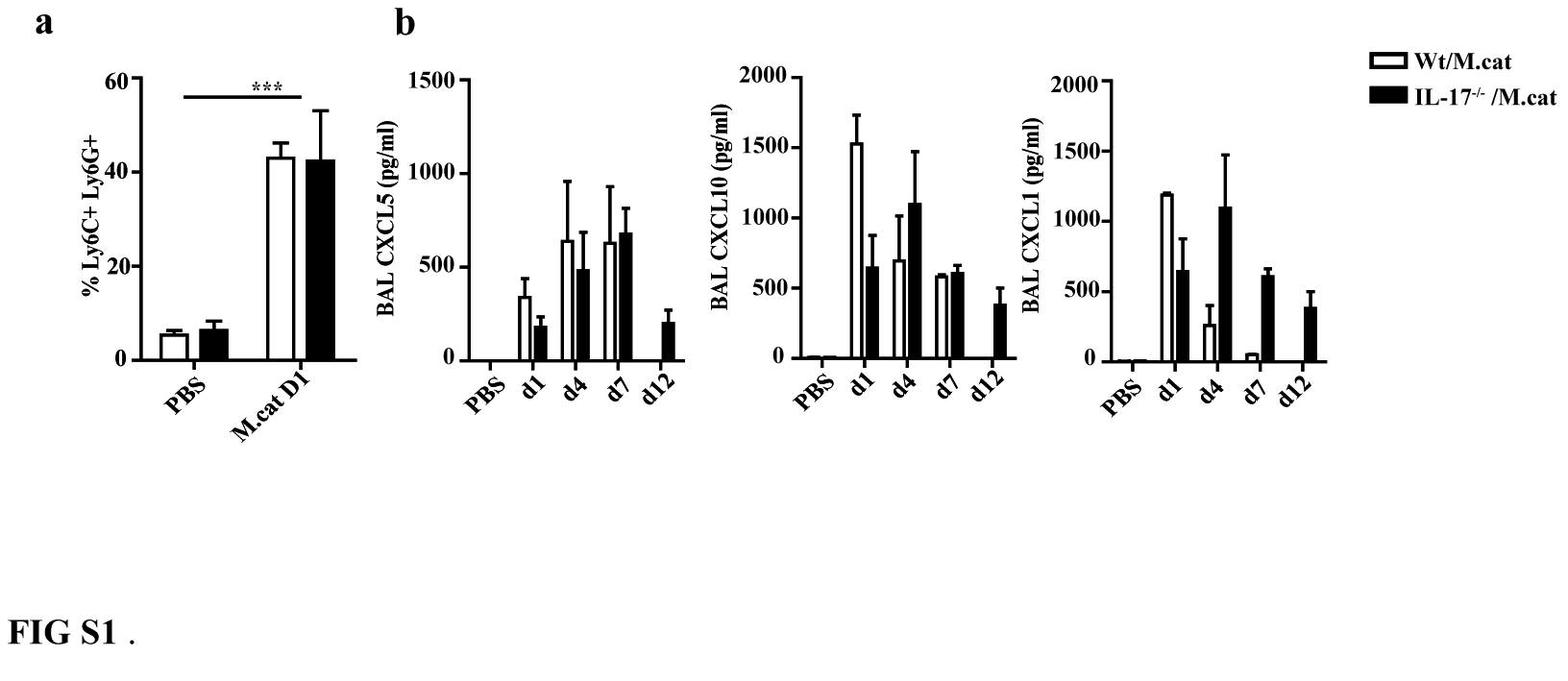

Supplement: Figure S1 — Early neutrophil influx induced by Moraxella catarrhalis is independent of IL-17. WT and IL-17AF−/− mice were infected (i.n.) with 2 × 10^8^ CFU M. catarrhalis. BALF and lungs were harvested at indicated time points. (A) Identification of BALF neutrophils by flow cytometry. Neutrophils were stained with Ly6C+Iy6G+ cells on day one after infection. (B) Amounts of CXCL1, CXCL5, and CXCL10 chemokines in BALF, n = 5 mice per group. Two independent experiments were performed ***P = 0.001, **P = 0.01, and *P = 0.05 (one-way ANOVA). BALF, BAL fluid; ANOVA, analysis of variance. [file Image_1.TIF]

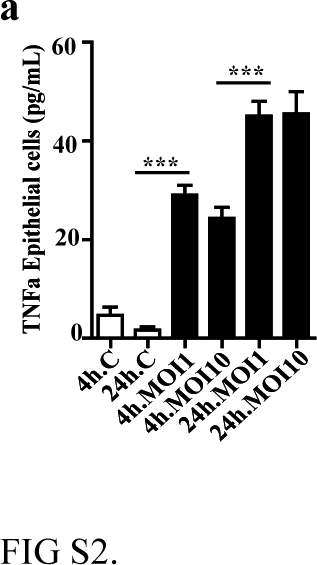

Supplement: Figure S2 — Tracheal epithelial cells produce TNF-a upon Moraxella catarrhalis infection. Primary tracheal cells from C57BL/6 mice were cultured and left untreated or infected with indicated MOI of M. catarrhalis. Culture supernatants were taken at indicated time-points and MOI and analyzed for secretion of TNF-α. Data represent two independent experiments with triplicates for each treatment condition. ***P = 0.001, **P = 0.01, and *P = 0.05 (one-way ANOVA). ANOVA, analysis of variance. [file Image_2.TIF]

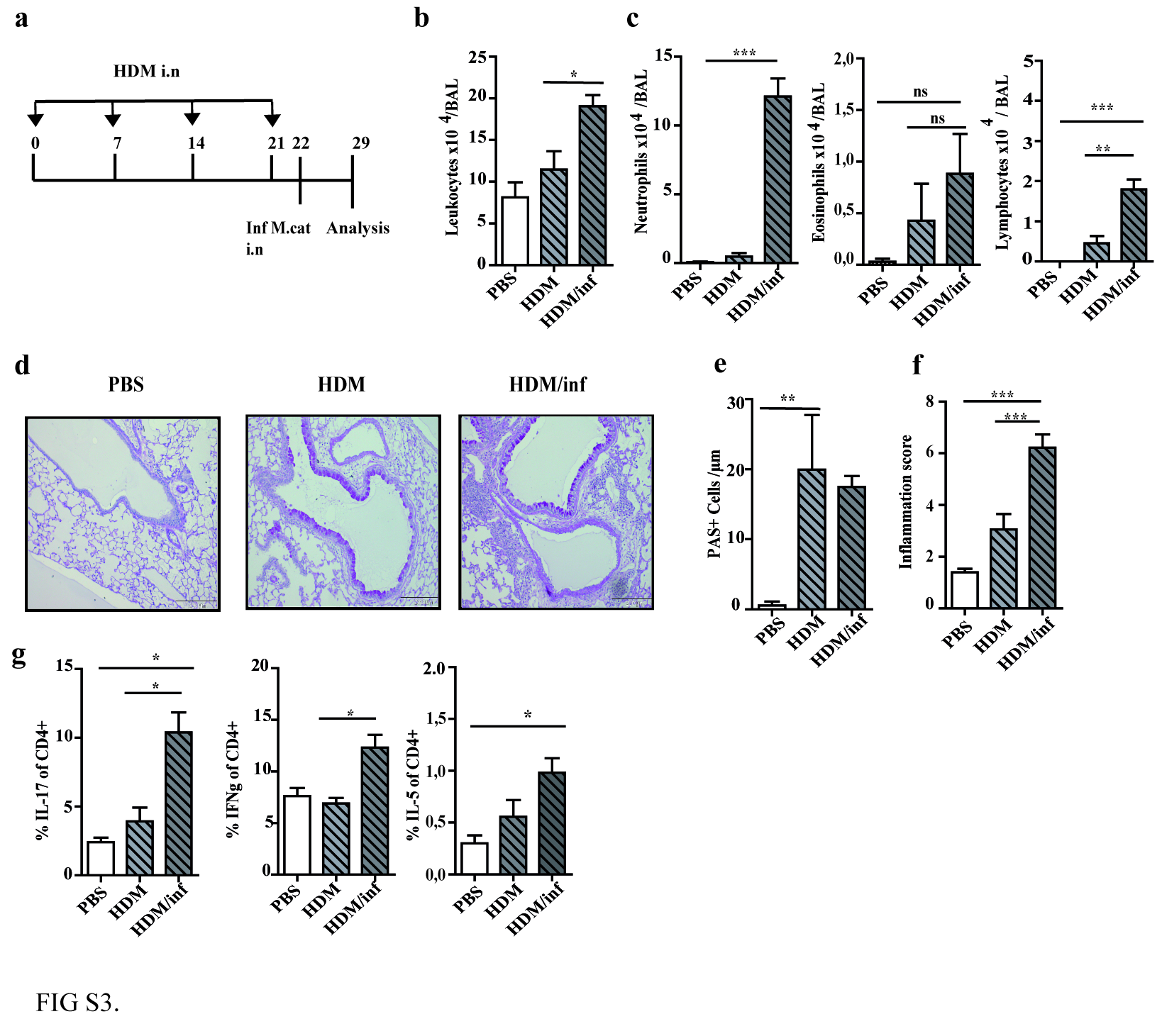

Supplement: Figure S3 — Moraxella catarrhalis exacerbates established HDM-allergic inflammation. C57BL/6 animals were infected (i.n.) with 2 × 10^8^ CFU M. catarrhalis after the last HDM exposure at day 22. BAL and lungs were analyzed at day 29. (A) Protocol. (B) Total cell counts in BALF. (C) Differential cell counts. (D) Representative periodic acid Schiff-stained airways. (E) Goblet cell counts in lung tissues. (F) Inflammation score. (G) Intracellular staining of lung CD4+ T cells for IL-17A, IFN-γ, and IL-5. Data were from two independent experiments (n = 8 mice per group). ***P = 0.001, **P = 0.01, and *P = 0.05 (one-one-way ANOVA). HDM, house dust mite; BAL, bronchoaleveolar lavage; BALF, BAL fluid; ANOVA, analysis of variance. [file Image_3.TIF]

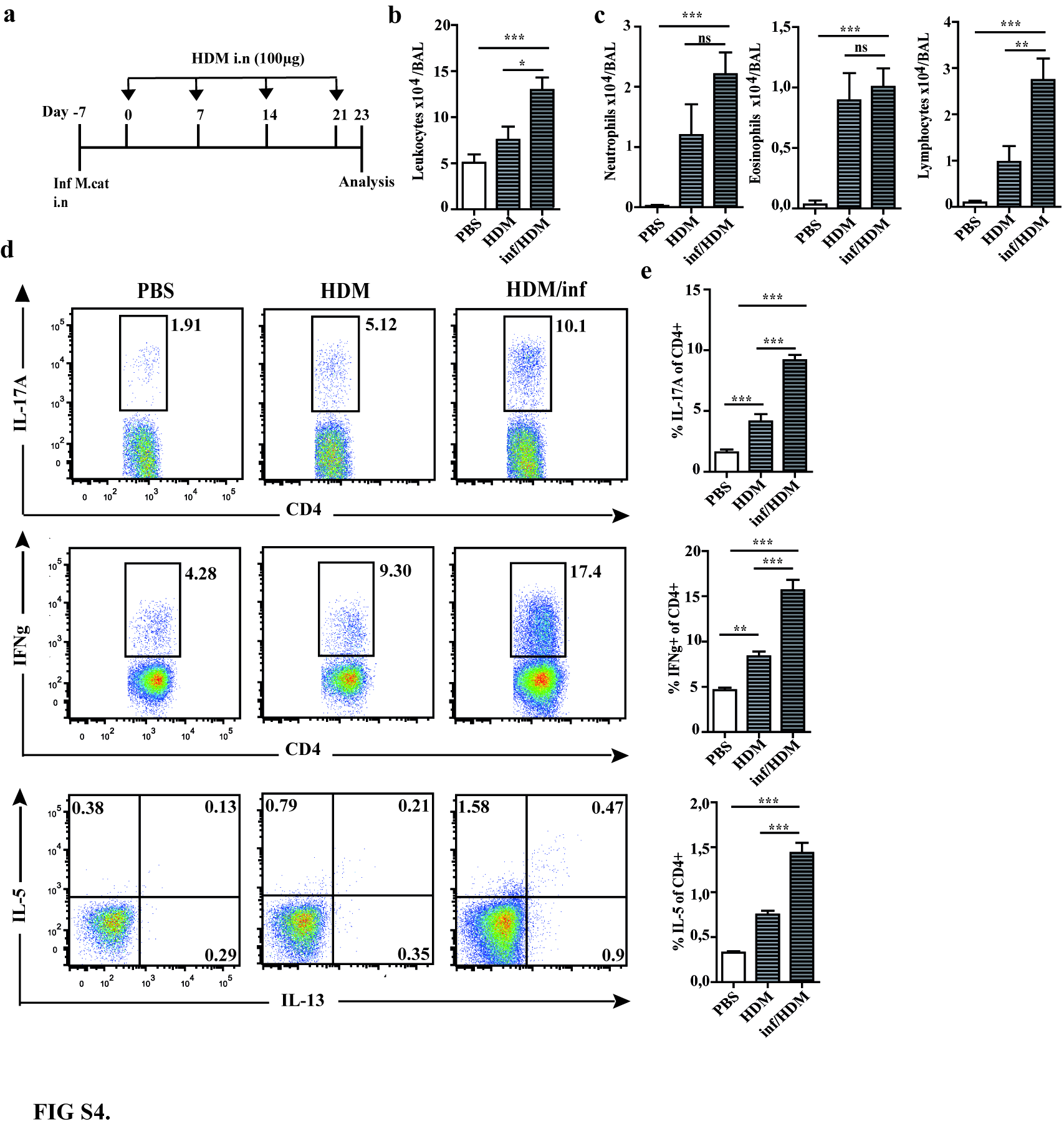

Supplement: Figure S4 — Moraxella catarrhalis infection prior to HDM exposure enhances pulmonary T-cell responses. C57BL/6 animals were infected (i.n.) with 2 × 10^8^ CFU M. catarrhalis. Seven days prior to HDM exposure. BAL and lungs were analyzed at day 23. (A) Protocol. (B) Total cell counts in BALF. (C) Differential cell counts. (D) Representative FACs analysis of pulmonary CD4+ T cells stained for IL-17A, IFN-γ and IL-5/IL-13. (E) Statistical distribution of T-cell populations (eight animals) corresponding to the analysis in D. Data were from two independent experiments, n = 4 mice per group. ***P = 0.001, **P = 0.01, and *P = 0.05 (one-way ANOVA). HDM, house dust mite; BAL, bronchoaleveolar lavage; BALF, BAL fluid; ANOVA, analysis of variance. [file Image_4.TIF]

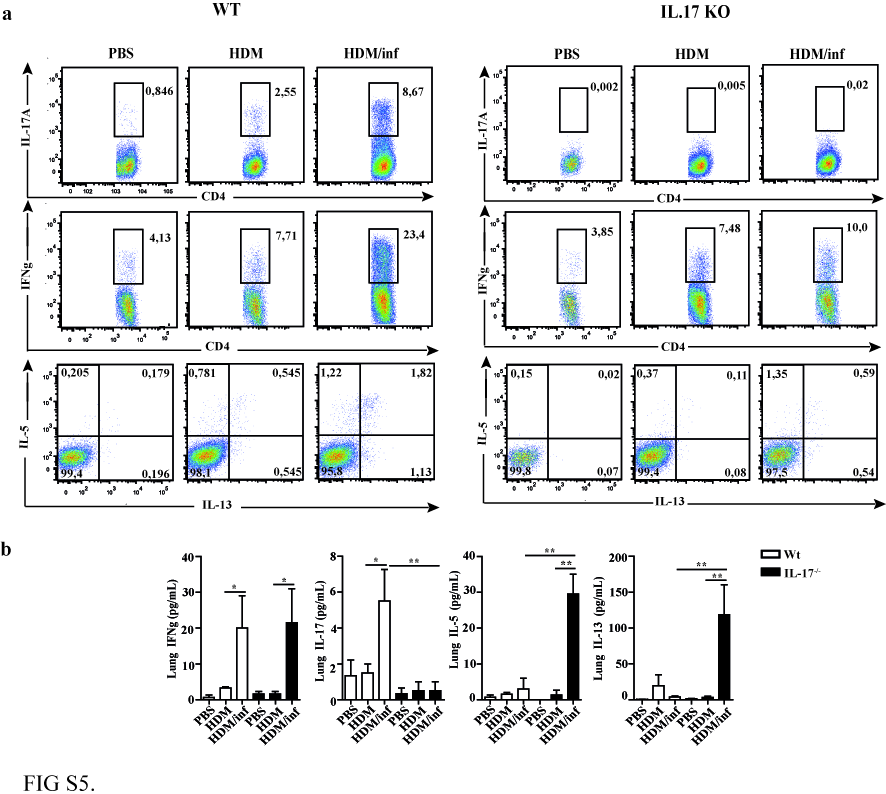

Supplement: Figure S5 — Analysis of cytokines in the lung of mice infected with Moraxella catarrhalis during HDM exposure. C57BL/6 and IL-17 KO animals were infected (i.n.) with 2 × 10^8^ CFU M. catarrhalis after the second HDM exposure. Lungs were analyzed at day 23. (A) Intracellular staining of lung CD4+ T cells was performed for IL-17A, IFN-γ, IL-5, and IL-13. Dot plots are representative of three similar experiments. (B) Amounts of IFN-γ, IL-17, IL-5, and IL-13 were measured by ELISA in lung homogenates. Data were from three independent experiments (n = 8 mice per group). **P = 0.01 and *P = 0.05 (one-way ANOVA). HDM, house dust mite; ANOVA, analysis of variance. [file Image_5.TIF]

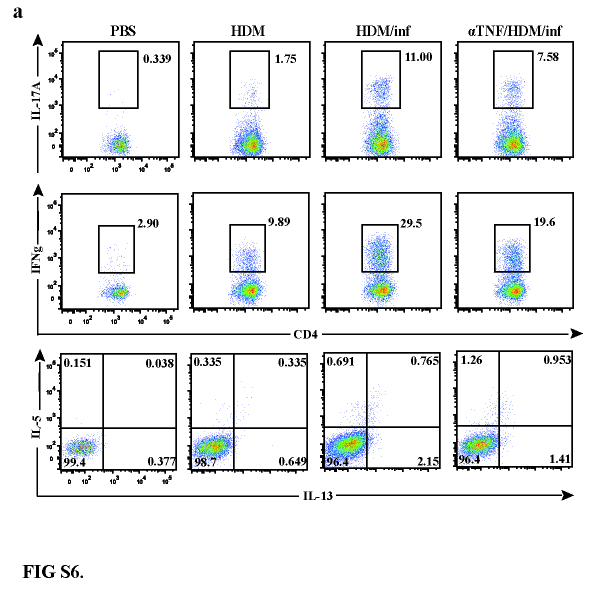

Supplement: Figure S6 — Effects of anti-TNF-α on inflammatory cytokines production in M. catarrhalis infected, HDM allergic mice. C57BL/6 animals were infected (i.n.) with M. catarrhalis (2 × 10^8^ CFU) after the second HDM exposure. Mice were treated i.p. with 100-µg anti-TNF-α or IgG1 control mAb, 4 h before infection with M. catarrhalis. Lung lymphocytes were analyzed at day 23. (A) Intracellular staining of lung CD4+ T cells for IL-17A, IFN-γ, IL-5, and IL-13. Data were from two independent experiments (n = 16 mice per group). ***P = 0.001, **P = 0.01, and *P = 0.05 (one-way ANOVA). HDM, house dust mite; ANOVA, analysis of variance. [file Image_6.TIF]
